# Metabolism of α-PHP and α-PHPP in humans and the effects of alkyl chain lengths on the metabolism of α-pyrrolidinophenone-type designer drugs

**DOI:** 10.1007/s11419-018-0428-7

**Published:** 2018-05-28

**Authors:** Shuntaro Matsuta, Noriaki Shima, Hidenao Kakehashi, Hiroe Kamata, Shihoko Nakano, Keiko Sasaki, Tooru Kamata, Hiroshi Nishioka, Akihiro Miki, Kei Zaitsu, Hitoshi Tsuchihashi, Munehiro Katagi

**Affiliations:** 1 Forensic Science Laboratory, Osaka Prefectural Police H.Q., 1-3-18 Hommachi, Chuo-ku, Osaka, 541-0053 Japan; 2 Department of Legal Medicine and Bioethics, Graduate School of Medicine, Nagoya University, 65 Tsurumai-cho, Showa-ku, Nagoya, 466-8550 Japan

**Keywords:** α-PHP and α-PHPP, α-Pyrrolidinophenone in vivo metabolism, Quantification of metabolites in urine specimen, LC–MS/MS, Alkyl chain length

## Abstract

**Purpose:**

This study aims to investigate the urinary metabolites of two common α-pyrrolidinophenones (PPs), α-pyrrolidinohexiophenone (α-PHP) and α-pyrrolidinoheptanophenone (α-PHPP). This report also aims to discuss the effects of alkyl chain lengths on the metabolism of PPs.

**Methods:**

Urinary metabolites of α-PHP and α-PHPP have been investigated by analyzing urine samples from their users (*n* = 13 each) by liquid chromatography–high-resolution tandem mass spectrometry using reference standards of the metabolites synthesized in our laboratory.

**Results and conclusions:**

For both drugs, metabolites via reduction of the keto moiety (1-OH metabolites) and via oxidation of the pyrrolidine ring (2″-oxo metabolites) were identified, and those via oxidation of the terminal (ω) or penultimate (ω-1) positions of the alkyl chain were tentatively identified. Quantitative analysis indicated oxidation of the pyrrolidine ring to be the major metabolic pathway for α-PHP (side chain R: hexyl), but ω or ω-1 oxidation was the major metabolic pathway for α-PHPP (R: heptyl). Comparison of their metabolic profiles with those of analogs with a longer or shorter side chain (studied previously for R: butyl, pentyl, and octyl) revealed that the alkyl chain length strongly influences the metabolic pathway. In addition, to the best of our knowledge, this is the first report describing the quantification of metabolites of α-PHP and α-PHPP in authentic urine specimens collected from the users using their reference standards synthesized.

**Electronic supplementary material:**

The online version of this article (10.1007/s11419-018-0428-7) contains supplementary material, which is available to authorized users.

## Introduction

α-Pyrrolidinophenones (PPs) are a class of cathinone-type designer drugs that are β-keto analogs of amphetamine (AP) with the amine substituted by a pyrrolidine. PPs include α-pyrrolidinobutiophenone (α-PBP), α-pyrrolidinopentiophenone (α-PVP), 4′-methyl-α-pyrrolidinopentiophenone (pyrovalerone), 3,4-methylenedioxypyrovalerone (MDPV), α-pyrrolidinohexiophenone (α-PHP), α-pyrrolidinoheptanophenone (α-PHPP), and α-pyrrolidinooctanophenone (α-POP). Because PPs have the structure of AP in the skeleton, some PPs induce central nervous system stimulant action, hyperactivity, and bizarre behavior due to reuptake inhibition of dopamine D_1_ and D_2_ and norepinephrine receptors [1–4].

Since PPs first appeared in 1996 as alternative drugs for APs, many new analogs have appeared and spread throughout the world. As a result, an increasing number of serious poisonings, traffic accidents, violent assaults, and even murders under the influence of PPs have been reported; this resulted in serious social problems in many countries. In addition to methamphetamine (MA), which has been the most prevalent illicit drug for decades in Japan, PPs, especially α-PVP, have been intensively used since the 2000s as MA-substitutes. α-PVP was banned in Japan as a narcotic drug in 2013 and the other PPs have comprehensively been scheduled alongside prohibiting their possession and use. However, even today, many kinds of PPs are still being sold and abused on the street. There is thus a pressing need to establish an effective analytical procedure to prove the use of PPs by detecting such drugs and their metabolites in urine.

In such forensic urine analysis, the detection of relevant metabolites in addition to the parent drug is important for indisputably proving ingestion of the drug. Several studies have been reported on PPs, including their instability in air [5], structural elucidation of newly encountered analogs [6, 7], and their qualitative [8, 9] and quantitative [10] analyses.

We have previously investigated and reported on the metabolites of α-PBP [11], α-PVP [12], and α-POP [13] in urine specimens from drug users. For α-PBP and α-PVP, it was elucidated that: (1) reduction of the carbonyl moiety and oxidation of the 2″-position of the pyrrolidine ring were the major metabolic pathways in human; (2) in these pathways, reduction of the carbonyl moiety is more predominant for α-PBP when compared with α-PVP; and (3) for α-PBP, notable imbalance in the ratio of diastereomers was observed for the metabolites generated by the reduction of the carbonyl moiety. However, the metabolism of α-POP significantly differed from those of α-PBP and α-PVP. Only trace amounts of such metabolites were detected for α-POP; instead, high abundance of metabolites derived from the oxidation of the terminal (ω) and penultimate (ω-1) positions of the alkyl chain were detected. These results suggested that the lengths of the alkyl chains have a significant influence on the metabolism of PPs.

There have been a few studies that investigated in silico [14] or in vitro [15–17] metabolism of PPs that have a phenyl group without substituents. Also, a few previous studies provided mass spectral elucidations of possible urinary metabolites of α-PHP [18] and α-PHPP [19], though these reports lack their identification by the reference standards of the candidate metabolites and quantification in authentic human urine specimens.

**Fig. 1:**
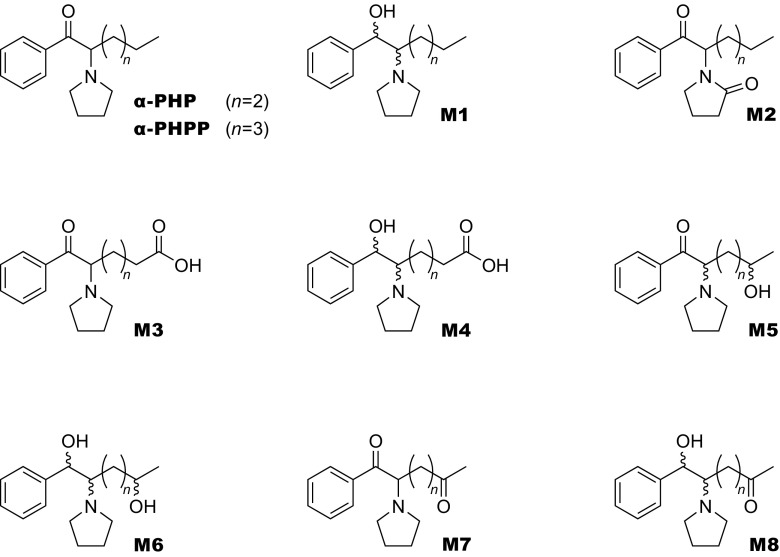
Structures of expected urinary metabolites of α-PHP and α-PHPP in humans

In this study, to further investigate the effects of alkyl chain lengths of PPs, we carefully determined the metabolites of α-PHP and α-PHPP in urine collected from the suspected users. We synthesized reference standards of the expected candidate metabolites produced by the reduction of the carbonyl moiety and the oxidation of 2″-position of the pyroridine ring (M1 and M2 shown in Fig. 1). The identification and quantification of these metabolites have been achieved using the synthesized reference standards to carefully investigate the metabolisms of our target drugs. The effects of the alkyl chain lengths on the metabolism of PPs is also discussed comprehensively based on the urinary elimination profiles of α-PHP and α-PHPP revealed here, in addition to those of α-PBP, α-PVP, and α-POP reported in our previous papers.

## Materials and methods

### Reagents

α-PHP, α-PHPP, 1-phenyl-2-(pyrrolidin-1-yl)hexan-1-ol (α-PHP-M1), 1-phenyl-2-(pyrrolidin-1-yl)heptan-1-ol (α-PHPP-M1), α-(2″-oxo-pyrrolidino)hexanophenone (α-PHP-M2), and α-(2″-oxo-pyrrolidino)heptanophenone (α-PHPP-M2) were synthesized in our laboratory and ensured as > 98% pure by ^1^H nuclear magnetic resonance (NMR) spectroscopy and quadrupole time-of-flight mass spectrometry (QTOF-MS) by direct flow injection (the procedure of chemical syntheses and analytical data for the confirmation of synthesized standards is summarized in supplemental material 1). Dibenzylamine, used as an internal standard (IS), was obtained from Wako Pure Chemical Industries (Osaka, Japan). Stock standard solutions of all analytes were prepared in methanol (MeOH) and diluted to appropriate concentrations with distilled water immediately prior to use. Distilled water and liquid chromatography–mass spectrometry (LC–MS)-grade MeOH were used throughout the experiments. All other reagents and chemicals used were of analytical grade or better quality, obtained from Wako Pure Chemical Industries.

### Urine specimens

Urine specimens analyzed here had been submitted to our laboratory for forensic drug analysis. The specimens were collected from drug users who possessed and admitted to the use of a powder form drug that was confirmed to be α-PHP or α-PHPP (13 subjects each), and were stored at − 20 °C until analysis. No exact and reliable information was available on the amount of drug used or the duration between drug ingestion and urine sampling. Drug-free urine used as blank urine was obtained from Golden West Biologicals (Temecula, CA, USA).

### Sample preparation

To 100-μL aliquots of urine samples, 100 μL of dibenzylamine (IS) aqueous solution (100 ng/mL), and 600 μL of MeOH were successively added and vortex-mixed for 1 min. The mixtures were centrifuged for 10 min at 7000 *g*, and the supernatants were transferred to a stoppered glass test tube and evaporated to dryness under nitrogen stream at 50 °C. The residues were each dissolved in 100 μL of distilled water. After filtration through a 0.22-μm membrane filter, 5-μL aliquots were used for LC–tandem MS (LC–MS/MS) analysis (see validation data summarized in supplemental material 2).

In quantitative analysis, samples were diluted with blank urine as needed prior to sample preparation.

### Instruments

#### NMR spectroscopy

NMR spectra of the synthesized compounds were acquired on a JNM-ECS 400 FT NMR system (JEOL Resonance, Tokyo, Japan) in CDCl_3_ containing tetramethylsilane as the IS.

#### LC–MS/MS

Quantitative analysis was performed on a Prominence Series ultra-fast liquid chromatography (UFLC) system (Shimadzu, Kyoto, Japan) linked to an API 3200 QTRAP hybrid triple quadrupole linear ion-trap mass spectrometer (QTrap; AB Sciex, Concord, ON, Canada) equipped with an electrospray ionization (ESI) interface. Qualitative analysis and identification were performed on the Prominence Series UFLC system linked to a Triple TOF 5600 hybrid QTOF mass spectrometer (AB Sciex) equipped with an ESI interface.

LC separation was carried out using an L-column 2 ODS semi-micro column (150 × 1.5-mm i.d., 5-μm particles; Chemicals Evaluation and Research Institute, Tokyo, Japan). The analytes were chromatographically separated by linear gradient elution with (A) 10 mM ammonium acetate buffer/MeOH (95:5, v/v) and (B) 10 mM ammonium acetate buffer/MeOH (5:95, v/v) at a flow rate of 0.1 mL/min and a column temperature of 40 °C. A gradient was applied starting from 0% B, and linearly increased to 100% over 20 min, and was held for 5 min. ESI-MS/MS was conducted in positive ion mode, and the protonated molecules were used as precursor ions. Analysis was performed in the selected reaction monitoring (SRM) mode for quantification by the 3200 QTrap, and in the high-resolution product ion scan (PIS) mode by using a Triple TOF 5600 for identification. Nitrogen was used as nebulizer and collision gas, and the declustering potential (DP) and collision energy (CE) were set at 30 V and 25 eV for PIS, respectively. The SRM parameters for each compound were set as shown in Table 1.

**Table 1:** Targeted compounds and their optimized selected reaction monitoring parameters for quantification by liquid chromatography–tandem mass spectrometry

Compound	Precursor ion (*m/z*)	Quantifier ion (*m/z*)	Dwell time (ms)	DP (V)	EP (V)	CEP (V)	CE (eV)	CXP (V)
α-PHP (unchanged)	246.2	91.1	30	40	8	16	25	3
M1-D1	248.2	230.2	30	25	8	16	19	3
M1-D2	248.2	91.1	30	25	8	16	30	3
M2	260.2	91.1	30	40	8	15	19	3
α-PHPP (unchanged)	260.2	91.1	30	40	8	16	25	3
M1-D1	262.2	244.2	30	25	8	16	19	3
M1-D2	262.2	91.1	30	25	8	16	30	3
M2	274.2	91.1	30	40	8	15	19	3
Dibenzylamine (IS)	198.1	91.1	30	31	7	12	27	4

*DP* declustering potential, *EP* entrance potential, *CEP* collision cell entrance potential, *CE* collision cell energy, *CXP* collision cell exit potential, *M* metabolite, *D* diastereomer, *IS* internal standard

## Results and discussion

### Metabolites M1 and M2 of α-PHP and α-PHPP

#### Identification and quantification of M1 and M2 in the users’ urine

**Fig. 2:**
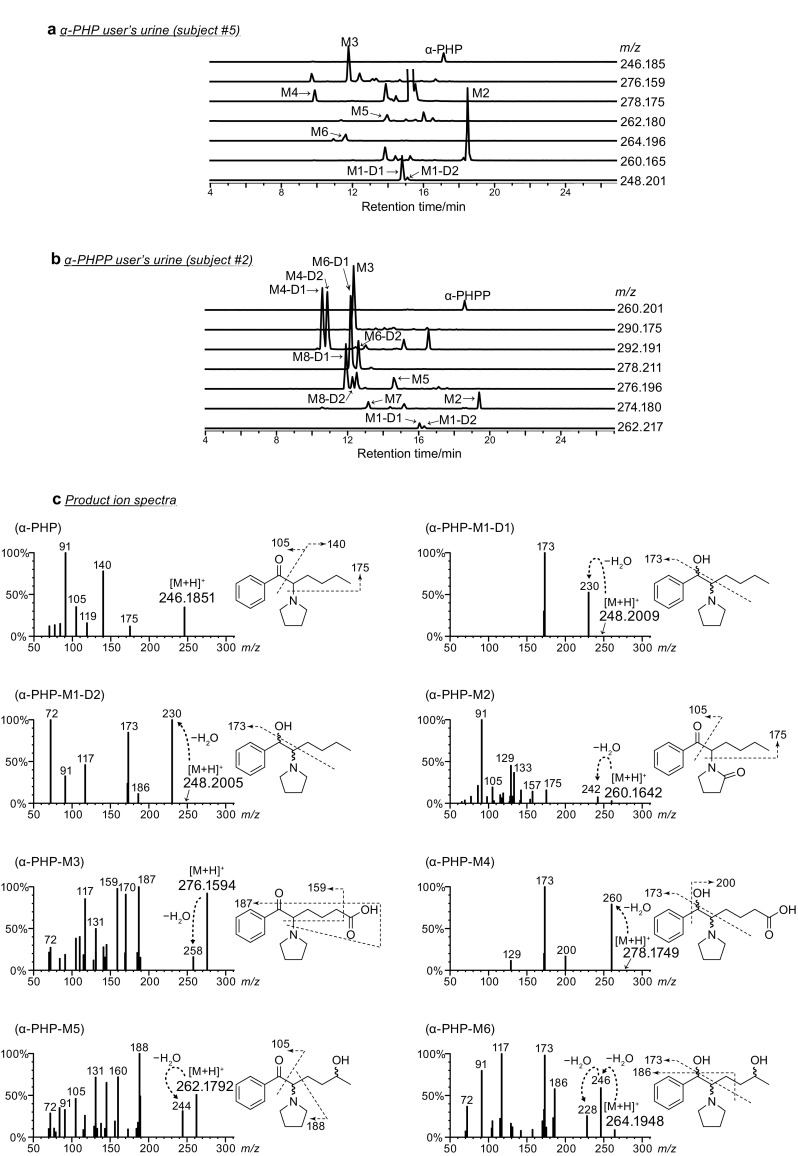

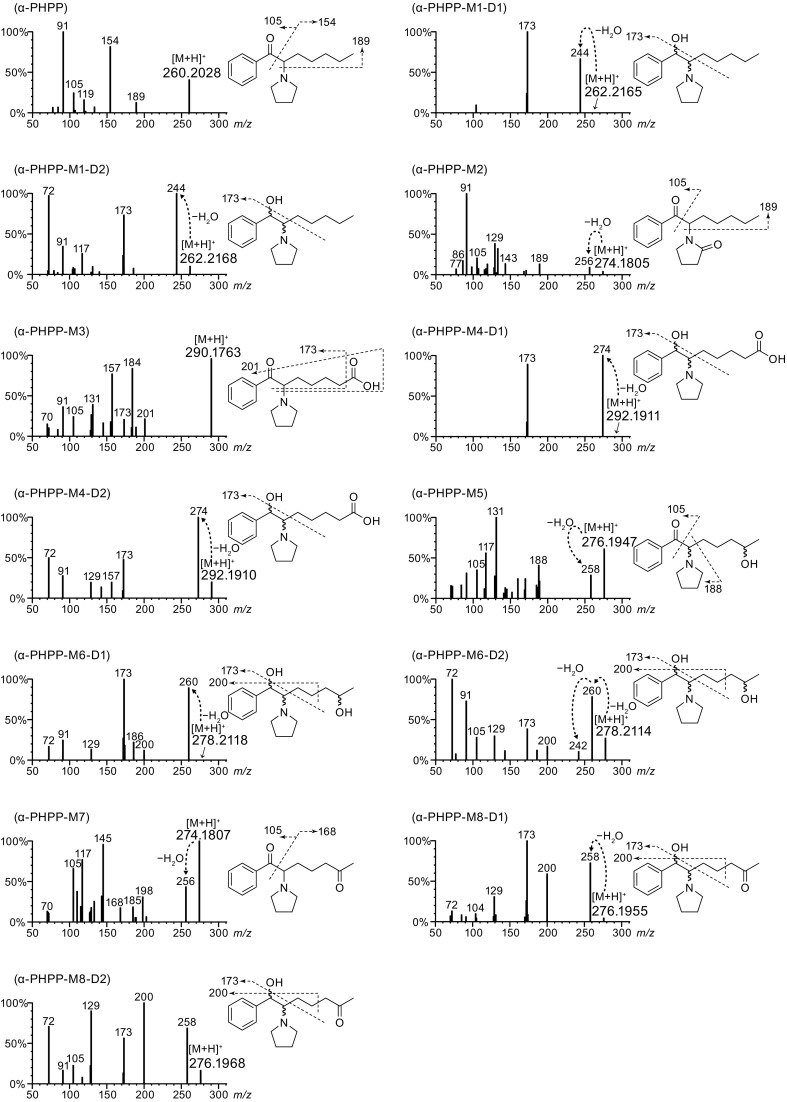
Typical extracted ion chromatograms from urine specimens of **a** an α-PHP user (subject 5 in Table 2) and **b** an α-PHPP user (subject 2 in Table 3); and **c** product ion mass spectra of α-PHP and α-PHPP, and their metabolites obtained by liquid chromatography–quadrupole time-of-flight tandem mass spectrometry. Mass labels other than protonated molecules are indicated as integers

Urine specimens from 13 each of the α-PHP and α-PHPP users were carefully analyzed by LC–QTOF-MS/MS procedure described in the experimental section. Urine specimens were analyzed without hydrolysis because phase II metabolism was found to be insignificant for PPs and complete hydrolysis was improbable for some of their phase II metabolites in our previous study [11–13]. Figure 2 presents typical extracted ion chromatograms and product ion spectra obtained by LC–QTOF-MS/MS from urine specimens of an α-PHP user and an α-PHPP user.

Detection of diastereomeric metabolites is expected for those containing plural chiral centers. For both α-PHP and α-PHPP, a pair of diastereomeric metabolites via reduction of the keto moiety (thereafter M1-D1 and M1-D2) and 2″-oxo metabolites (M2) were identified in all of the urine specimens, using reference standards newly synthesized in this study. Proton NMR spectroscopy data of M1-D1 and M1-D2 (data shown in supplemental material 1) are indicated for both drugs.

In our previous study, corresponding metabolites were also detected commonly for α-PBP, α-PVP, and α-POP [11–13], but their abundances appeared to vary among the PP analogs. The concentrations of the parent drugs and their above-mentioned metabolites in urine (13 each for α-PHP users and α-PHPP users) were quantitated by the method detailed in the experimental section. All validation data were satisfactory as shown in Table S1 (supplementary material 2). The results for α-PHP and α-PHPP users are listed in Tables 2 and 3, respectively.

**Table 2:** Urinary concentrations of α-PHP and its metabolites in α-PHP users’ urine specimens

Subject no.	Urinary concentration (ng/mL)			
	α-PHP	M1-D1	M1-D2	M2
1	5940	4300	427	6420
2	2980	2030	99.3	4860
3	697	290	34.9	830
4	519	178	24.4	348
5	471	1120	138	5920
6	298	198	19.0	186
7	218	432	29.6	2550
8	201	250	16.5	703
9	140	102	3.93	731
10	118	544	14.3	532
11	112	114	8.10	397
12	41.6	35.2	NQ	477
13	31.9	23.2	NQ	39.4

*NQ* not quantified

**Table 3:** Urinary concentrations of α-PHPP and its metabolites in α-PHPP users’ urine specimens

Subject no.	Urinary concentration (ng/mL)			
	α-PHPP	M1-D1	M1-D2	M2
1	4730	478	236	6370
2	4120	1550	1130	11600
3	777	666	382	843
4	728	97.3	56.6	412
5	563	23.3	14.6	1560
6	531	165	97.9	1500
7	317	22.1	10.5	787
8	309	191	87.9	1170
9	287	33.5	12.5	1270
10	224	31.4	12.8	362
11	144	45.2	19.3	547
12	129	8.41	4.58	444
13	113	7.05	3.71	137

The concentration ratios of M1 (the sum of diastereomers M1-D1 and M1-D2) to each parent drug were 1.34 ± 1.21 for α-PHP (*n* = 13) and 0.379 ± 0.387 for α-PHPP (*n* = 13; average ± standard deviation). Regarding the diastereomic selectivity, M1-D1 was found to be more abundant than M1-D2 for all the specimens tested here and for both drugs. The diastereomic ratios of M1-D1/M1 were 0.933 ± 0.033 (*n* = 13) for α-PHP and 0.659 ± 0.042 (*n* = 13) for α-PHPP. Thus, both the M1 ratio (to each parent drug) and the diastereomic ratio (M1-D1/M1) were higher for α-PHP than those for α-PHPP.

On the same topics investigated previously for α-PBP (R: butyl), α-PVP (R: pentyl), and α-POP (R: octyl), elongation of the side chain resulted in lower M1 ratios (to each parent drug) and lower diastereomic ratios (M1-D1/M1) [11–13].

For the 2″-oxo metabolites M2 generated by the oxidation at the 2″ position of the pyrrolidine ring, the ratios of M2 to each parent drug were 4.53 ± 4.46 (*n* = 13) for α-PHP and 2.47 ± 1.22 (*n* = 13) for α-PHPP (Tables 2 and 3). In our previous studies for α-PBP and α-PVP, 2″-oxo metabolites (M2) were detected in high abundance for the latter, which possesses a longer side chain (pentyl) [11, 12]. However, for α-PHP and α-PHPP investigated here, M2 was more abundant for the former, which has a shorter side chain (butyl). In addition, only a trace amount of M2 was detected for α-POP (octyl) in our separate study [13]. It was thus assumed that the relative abundance of the 2″-oxo metabolite M2 is greatest for α-PHP (R: hexyl), and decreases for PPs with a longer or shorter side chain.

**Fig. 3:**
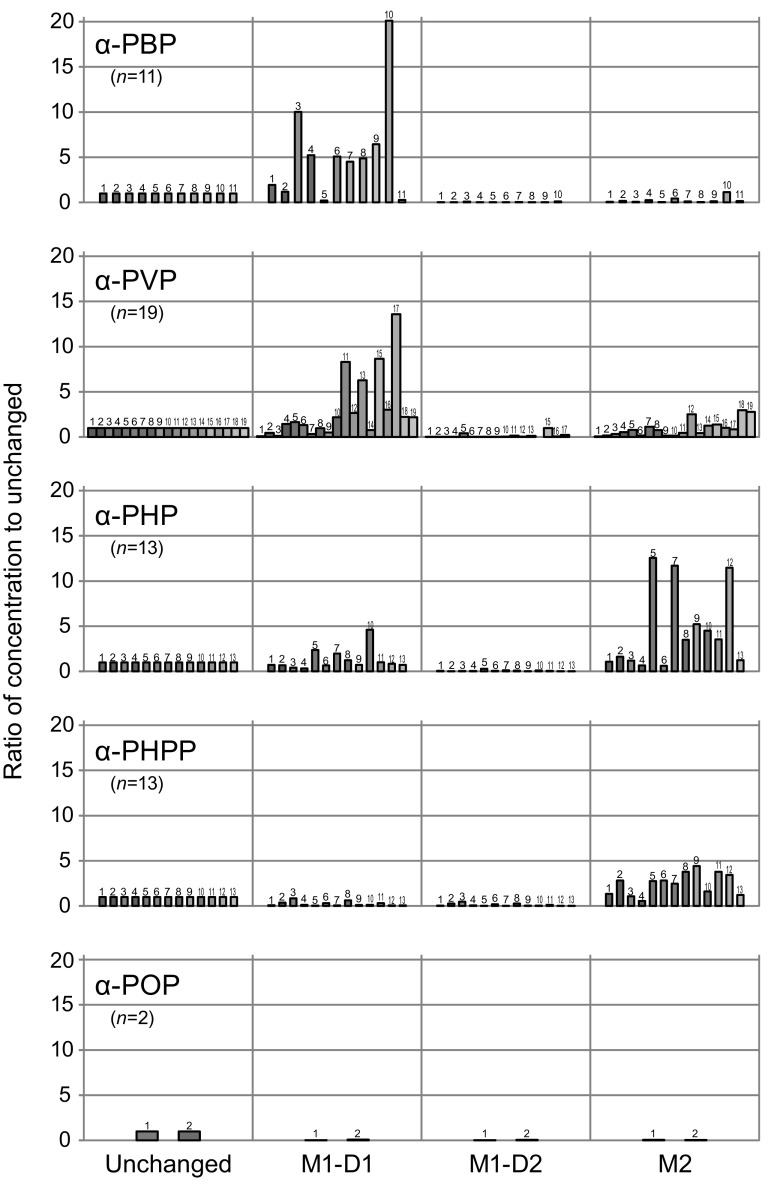
Individual concentration ratios of the metabolites M1-D1, M1-D2, and M2 to the corresponding parent drugs detected in α-PHP users’ (*n* = 13) and α-PHPP users’ (*n* = 13) urine specimens together with those in α-PBP (*n* = 11), α-PVP (*n* = 19), and α-POP (*n* = 2) users reported previously [11–13]. In the “unchanged” column, all bars show a ratio of 1

Figure 3 summarizes the individual concentration ratios of M1-D1, M1-D2, and M2 to the corresponding parent drugs detected in each of the 13 α-PHP users’ and α-PHPP users’ urine specimens tested here, as well as those for α-PBP (*n* = 11), α-PVP (*n* = 19), and α-POP (*n* = 2) reported previously [11–13], for comparison.

### Metabolites involving oxidation of the side chain

Because both M1 and M2 for α-PHP and α-PHPP decreased as the side chain lengthened, we next focused on alternative metabolisms, including aliphatic oxidation of the side chain, as previously reported for α-PVP [12] and α-POP [13]. Metabolites involving oxidation of the side chain were carefully investigated for urine specimens from five α-PHP users and six α-PHPP users (randomly selected), which were found to contain relatively abundant metabolites by LC–QTOF-MS, considering the analytical data previously obtained for α-PVP and α-POP. Typical analytical data obtained from an α-PHP user and an α-PHPP user are presented in Fig. 2a.

A carboxylic acid-type metabolite generated probably via the aliphatic hydroxylation of the ω position followed by its oxidation was putatively identified for both drugs (hereafter M3). Also, another carboxylic acid-type metabolite resulted by combination of the above-mentioned ω oxidation and reduction of the carbonyl moiety was also putatively identified for both drugs (hereafter M4). Although a pair of M4 diastereomers were detected for α-PHPP, only one M4 peak was detectable for α-PHP that also has two chiral centers in its molecule, probably due to their incomplete separation of the diastereomers. Similarly, two kinds of metabolites generated via the aliphatic hydroxylation in ω-1 position were putatively detected for both drugs (hereafter M5 and M6, see Figs. 1 and 2). For M6, which has three chiral centers (see Fig. 1), two peaks were detected for α-PHPP, but only one peak was observed for α-PHP, probably for the same reasons as mentioned above. In addition, for α-PHPP only, metabolites generated from M5 and M6 by further oxidation of the hydroxyl moiety at the ω-1 position into a carbonyl moiety (hereafter M7 and M8, respectively, see Figs. 1 and 2) were also putatively detected for all of the six urine specimens tested here (M8 was detected as diastereomers).

Metabolites M3 to M8 were putatively identified without using reference standards by comparing their mass spectra with those of corresponding α-POP metabolites [13] that were previously obtained using synthesized reference standards based on the facts as follows: (1) satisfactory confirmation of high-resolution mass spectra of protonated molecular ions, product ions containing an alkyl side chain (at *m/z* losing 14 or 28 from that of α-POP), and product ions that do not contain an alkyl side chain (at the same *m/z* as with α-POP); (2) the fragmentation patterns in the product ion spectra resembled those of α-POP, and (3) the pattern of LC chromatograms and the order of peaks are very similar to that of α-POP.

Moreover, possible metabolites generated by ring cleavage of M2 were tentatively detected for both α-PHP and α-PHPP (analytical data not shown), as previously reported for α-PVP by Tyrkkö [14], and for α-PHP by Paul [18]. However, it should be noted that such highly degraded metabolites have only limited importance as evidence for proving the ingestion of the drug because they no longer keep the basic skeleton characterizing the parent drug.

### Abundance of metabolites involving oxidation of the side chain

**Fig. 4:**
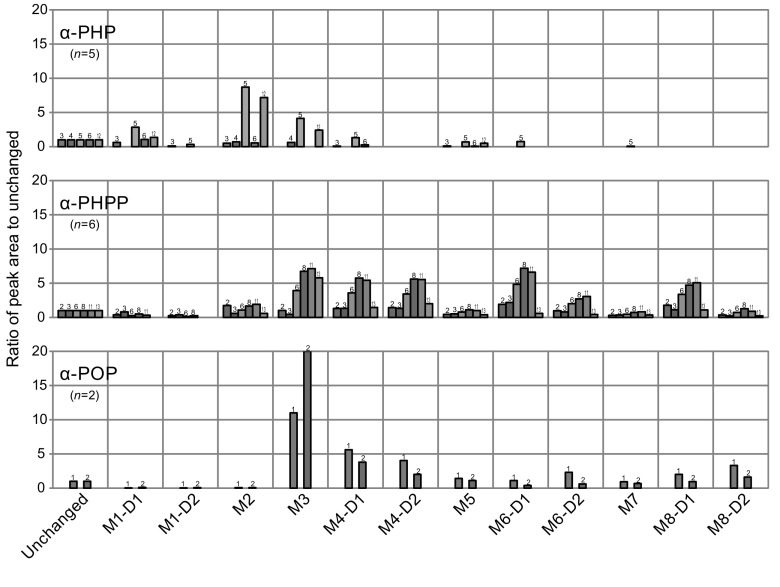
Peak area ratios against unchanged drugs for various metabolites detected in urine specimens from five α-PHP users and six α-PHPP users tested here, together with those for two α-POP users (quoted from our previous report [13])

Figure 4 summarizes the relative abundance of the metabolites M3–M8 involving oxidation of the side chain reported above, and those of M1 and M2 for comparison, detected in urine specimens from five α-PHP users and six α-PHPP users analyzed here, together with those of two α-POP users quoted from our previous report [13] for discussion. For these putatively identified metabolites, their relative area abundances (to the parent drug) were used for their relative amounts on the extracted ion chromatograms at the *m/z* of each protonated molecule. This substitution could be justified based on the agreeable proportionality that was observed between peak areas and concentrations among all of α-POP metabolites synthesized in our previous study [13].

For metabolites involving ω oxidation, M3 and a pair of M4 diastereomers (M4-D1 and M4-D2) were detected in all of the urine specimens tested for α-PHPP and α-POP. However, for α-PHP, although either or both M3 and M4-D1 were detectable in urine specimens tested here, M4-D2 was not detected in any of the specimens. All metabolites involving ω-1 oxidation (M5–M8, including diastereomer pairs of M6 and M8) were putatively detected in all urine specimens tested for α-PHPP and α-POP. For α-PHP, however, although M5 was detected in four specimens, M6-D1 and M7 were detected in only one specimen out of the five tested here.

For α-PHP, it was indicated that oxidation of the 2″ position of the pyrrolidine ring and that of the ω position of the side chain were the predominant metabolic pathways although remarkable individual variations existed. For α-PHPP, on the other hand, metabolism by aliphatic oxidation of the side chain at ω or ω-1 position was predominant over oxidation of the pyrrolidine ring (Fig. 4).

**Fig. 5:**
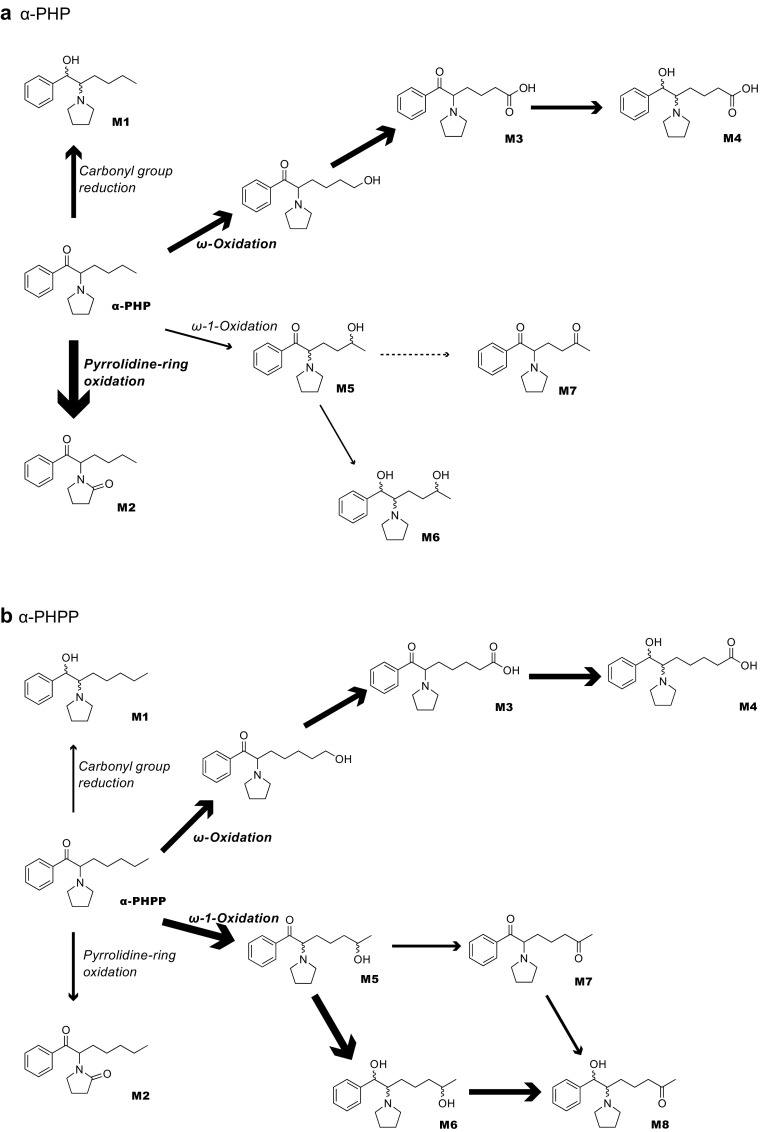
Proposed metabolic pathways of **a** α-PHP and **b** α-PHPP in humans

The metabolic pathways of α-PHP and α-PHPP suggested based on the above-mentioned analytical results are illustrated in Fig. 5.

### Effects of side chain lengths on the metabolic pathways

**Fig. 6:**
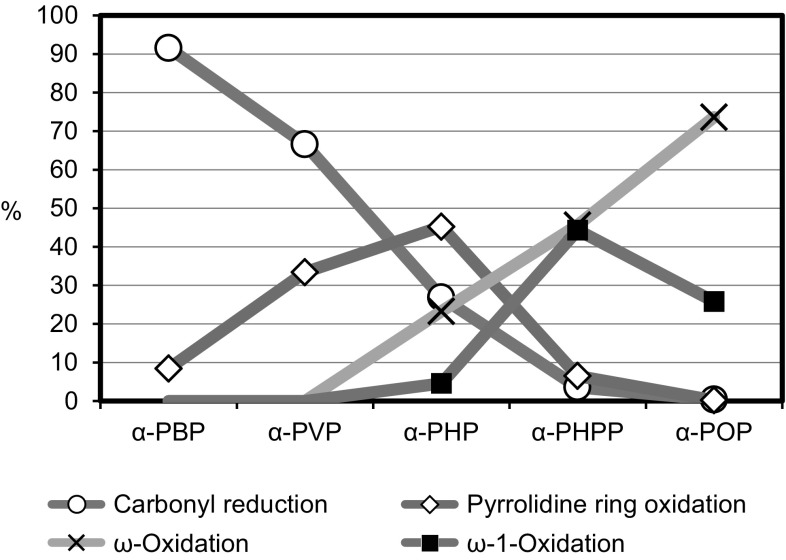
Averaged percentage composition of various relevant metabolites of PPs detected in the users’ urine specimens. Open cycle symbol, carbonyl reduction (M1-D1 + M1-D2); open diamond symbol, pyrrolidine ring oxidation (M2); cross symbol, ω oxidation (M3 + M4-D1 + M4-D2); solid square symbol, ω-1 oxidation (M5 + M6-D1 + M6-D2 + M7 + M8-D1 + M8-D2). Mean values were plotted

The above-mentioned results and our previous research for related PPs suggest that the side chain length have remarkable effects on the metabolism of PPs. In our previous studies for α-PBP, α-PVP, and α-POP [11–13], it was found that the shorter the aliphatic side chain, the higher the concentration ratio of M1 (to the parent drug) and the higher the diastereomic ratio (M1-D1/M1). These tendencies, (i.e., such effects of the side chain lengths on the metabolites M1) were also observed for α-PHP (R: hexyl) and α-PHPP (R: heptyl) investigated in this study. Thus, the order of concentration ratio of M1 to each parent drug as well as of the diastereomic ratio M1-D1/M1 is as follows: α-PBP > α-PVP > α-PHP > α-PHPP > α-POP. The effects on the diastereomic ratio are probably due to the stereoselectivity for the enzymatic reduction of the carbonyl moiety, which is deteriorated by the elongation of the side chain. Figure 6 illustrates the effects of side-chain lengths on the composition of various metabolites detected in the users’ urine specimens, prepared using the results for α-PHP (R: hexyl) and α-PHPP (R: heptyl) investigated here, and those of α-PBP (R: butyl), α-PVP (R: pentyl), and α-POP (R: octyl) in our previous reports [11–13].

As shown in Fig. 6, although reduction of the carbonyl moiety (to produce M1) is the overwhelmingly predominant metabolism for α-PBP, it decreases with elongation of the side chain. For the oxidation of pyrrolidine ring (to form the 2″-oxo metabolite M2), it was revealed that its relative abundance is highest for α-PHP (R: hexyl), and decreases with a longer or shorter side chain. For PPs with longer side chain, oxidation of the side chain at ω and ω-1 positions becomes more significant as the side chain is lengthened. The metabolite with ω oxidation (M3 and M4) were detected from α-PHP or longer, and those with ω-1 oxidation (M5–M8) were detectable from α-PHPP or longer. Most of the metabolites detected for α-POP were those involving aliphatic oxidation of the side chain, and only trace-levels of M1 and M2 were detectable for α-POP.

## Conclusions

As a result of the present study for the urinary metabolites of α-PHP and α-PHPP, their proposed metabolic pathways in humans include: (1) reduction of the keto group to the corresponding alcohol, (2) oxidation of the 2″ position of the pyrrolidine ring to the oxo moiety; (3) aliphatic oxidation at ω and/or ω-1 positions of the side chain to form the corresponding carboxylic acids, alcohols, and/or ketones; and (4) combinations of these pathways. In addition, comparison with analogs having a shorter or longer side chain revealed that the length of side chain significantly affects their metabolic pathways.

Synthetic cathinones, especially PPs addressed in this study, have been one of the most prevalent classes of drugs in the drug market, despite powerful administrative acts including comprehensive analog regulations. Among the growing variety of analogs, elongation of the side chain is the most rampant molecular manipulation in clandestine laboratories. The above-mentioned findings can provide useful information for metabolism studies as well as the establishment of analytical procedures, both in forensic toxicology and in drug administration, not only for cathinone analogs, but also for possible emerging drugs that may appear on the streets.

Finally, it should be pointed out that this is the first report describing the quantification of metabolites including their diastereomers of α-PHP and α-PHPP in authentic urine specimens collected from users using their reference standards synthesized in our laboratory.

## Electronic supplementary material

Below is the link to the electronic supplementary material.

Supplementary material 1 (PDF 269 kb)

Supplementary material 2 (PDF 171 kb)

## References

[1] Baumann MH, Ayestas MA, Partilla JS, Sink JR, Shulgin AT, Daley PF, Brandt SD, Rothman RB, Ruoho AE, Cozzi NV (2012). The designer methcathinone analogs, mephedrone and methylone, are substrates for monoamine transporters in brain tissue. Neuropsycopharmacology.

[2] Baumann MH, Partilla JS, Lehner KR, Thorndike EB, Hoffman AF, Holy M, Rothman RB, Goldberg SR, Lupica CR, Sitte HH, Brandt SD, Tella SR, Cozzi NV, Schindler CW (2013). Powerful cocaine-like actions of 3,4-methylenedioxypyrovalerone (MDPV), a principal constituent of psychoactive ‘bath salts’ products. Neuropsycopharmacology.

[3] Kakizaki A, Tanaka S, Numazawa S (2014). New recreational drug 1-phenyl-2-(1-pyrrolidinyl)-1-pentanone (α-PVP) activates central nervous system via dopaminergic neuron. J Toxicol Sci.

[4] Marusich JA, Antonazzo KR, Willey JL, Blough BE, Partilla JS, Baumann MH (2014). Pharmacology of novel synthesic stimulants structurally related to the “bath salts” constituent 3,4-methylenedioxypyrovalerone (MDPV). Neuropharmacology.

[5] Tsujikawa K, Yamamuro T, Kuwayama K, Kanamori T, Iwata YT, Inoue H (2015). Instability of the hydrochloride salts of cathinone derivatives in air. Forensic Sci Int.

[6] Matsuta S, Katagi M, Nishioka H, Kamata H, Sasaki K, Shima N, Kamata T, Miki A, Tatsuno M, Zaitsu K, Tsuboi K, Tsuchihashi H, Suzuki K (2014). Structural characterization of cathinone-type designer drugs by EI mass spectrometry (in Japanese with English abstract). Jpn J Forensic Sci Technol.

[7] Matsuta S, Kakehashi H, Nakano S, Shima N, Kamata T, Nishioka H, Miki A, Sakamoto Y, Miyagawa H, Kusano M, Zaitsu K, Tsuchihashi H, Katagi M (2017). Comprehensive analysis and structural estimation of synthetic cathinones using GC–MS/MS (in Japanese with English abstract). Jpn J Forensic Sci Technol.

[8] Concheiro M, Castaneto M, Kronstrand R, Huestis MA (2015). Simultaneous determination of 40 novel psychoactive stimulants in urine by liquid chromatography-high resolution mass spectrometry and library matching. J Chromatogr A.

[9] Waters B, Ikematsu N, Hara K, Fujii H, Tokuyasu T, Tanaka M, Matsusue A, Kashiwagi M, Kubo S (2016). GC–PCI–MS/MS and LC–ESI–MS/MS databases for the detection of 104 psychotropic compounds (synthetic cannabinoids, synthetic cathinones, phenethylamine derivatives). Leg Med.

[10] Sugie K, Akutsu M, Saito K (2016). Quantitative analysis of NPS (new psychoactive substance) containing α-PVP by direct analysis in real time (DART)-TOF-MS with micro syringe injection (in Japanese with English abstract). Bunseki Kagaku.

[11] Matsuta S, Shima N, Kamata H, Kakehashi H, Nakano S, Sasaki K, Kamata T, Nishioka H, Miki A, Katagi M, Zaitsu K, Sato T, Tsuchihashi H, Suzuki K (2015). Metabolism of the designer drug α-pyrrolidinobutiophenone (α-PBP) in humans: identification and quantification of the phase I metabolites in urine. Forensic Sci Int.

[12] Shima N, Katagi M, Kamata H, Matsuta S, Sasaki K, Kamata T, Nishioka H, Miki A, Tatsuno M, Zaitsu K, Ishii A, Sato T, Tsuchihashi H, Suzuki K (2014). Metabolism of the newly encountered designer drug α-pyrrolidinovalerophenone in humans: identification of urinary metabolites. Forensic Toxicol.

[13] Shima N, Kakehashi H, Matsuta S, Kamata H, Kamata T, Nishioka H, Zaitsu K, Sato T, Miki A, Katagi M, Tsuchihashi H (2015). Urinary excretion and metabolism of the α-pyrrolidinophenone designer drug 1-phenyl-2-(pyrrolidin-1-yl)octan-1-one (PV9) in humans. Forensic Toxicol.

[14] Tyrkkö E, Pelander A, Ketola RA, Ojanperä I (2013). In silico and in vitro metabolism studies support identification of designer drugs in human urine by liquid chromatography/quadrupole-time-of-flight mass spectrometry. Anal Bioanal Chem.

[15] Maurer HH, Kraemer T, Springer D, Staack RF (2004). Chemistry, pharmacology, toxicology, and hepatic metabolism of designer drugs of amphetamine (ecstasy), piperazine, and pyrrolidinophenone types: a synopsis. Ther Drug Monit.

[16] Chen X (2015). Simultaneous determination of four designer drugs and their major metabolites by liquid chromatography–mass spectrometry. J Chromatogr B.

[17] Negreira N, Erratico C, Kosjek T, van Nuijs ALN, Heath E, Neels H, Covaci A (2015). In vitro Phase I and Phase II metabolism of α-pyrrolidinovalerone (α-PVP), methylenedioxypyrovalerone (MDPV) and methedrone by human liver microsomes and human liver cytosol. Anal Bioanal Chem.

[18] Paul M, Bleicher S, Guber S, Ippisch J, Polettini A, Schultis W (2015). Identification of phase I and II metabolites of new designer drug α-pyrrrolidinohexiophenone (α-PHP) in human urine by liquid chromatography quadrupole time-of-flight mass spectrometry (LC–QTOF-MS). J Mass Spectrom.

[19] Swortwood MJ, Ellefsen KN, Wohlfarth A, Diao X, Concheiro-Guisan M, Kronstrand R, Huestis MA (2016). First metabolic profile of PV8, a novel synthetic cathinone, in human hepatocytes and urine by high-resolution mass spectrometry. Anal Bioanal Chem.

