# Supplementary material for: Metabolism of α-PHP and α-PHPP in humans and the effects of alkyl chain lengths on the metabolism of α-pyrrolidinophenone-type designer drugs
Source: Forensic Toxicol. 2018 May 28;36(2):486–97. doi: 10.1007/s11419-018-0428-7 (PMC6002437; doi:10.1007/s11419-018-0428-7)
Supplement: Supplementary file 1 — Supplementary material 1 (PDF 269 kb) [file 11419_2018_428_MOESM1_ESM.pdf]

Supplementary material 1: chemical synthesis of analytical standards

α-PHP, α-PHPP, α-PHP-M1, α-PHPP-M1, α-PHP-M2, and α-PHPP-M2 were synthesized in our laboratory based on our previously published methods [11–13], as follows:

α-PHP

To a methylene chrolide (CH_2Cl_2) solution of hexanophenone (500 mg), one drop of bromine was added, and this was stirred for 5 min to initiate the reaction. An equivalent of bromine in CH_2Cl_2 solution was added and stirred over 10 min. The solvent was then removed under vacuum to yield 2-bromo-hexanophenone.

To a solution of 2-bromo-hexanophenone (200 mg) in tetrahydrofuran (THF) was added dropwise pyrrolidine in THF, and the mixture was stirred at room temperature overnight. The reaction mixture was treated with 10 % HCl to make it acidic and was then washed with diethyl ether. The aqueous layer was then made basic with 10 % Na_2CO_3 and extracted with ethyl acetate. The organic extract was washed with brine, dried over anhydrous Na_2SO_4, and evaporated to give crude α-PHP as pale yellow oil. Finally, 10 % HCl MeOH solution was added dropwise to the oil. After removal of the solvent, the crude hydrochloride salt was purified by recrystallization from diethyl ether/2-propanol.

1-Phenyl-2-(pyrroridin-1-yl)hexane-1-ol (α-PHP-M1)

To a solution of α-PHP (150 mg) in ethanol at 60 °C was added sodium tetrahydroborate, and the mixture was stirred at 60 °C for 1 h. The reaction mixture was evaporated under vacuum, and the residue was treated with 10 % HCl aqueous solution to make it acidic and was then washed with diethyl ether. The aqueous layer was then made basic with 10 % Na_2CO_3 and extracted with ethyl acetate. The organic extract was washed with brine, dried over anhydrous Na_2SO_4, and evaporated to give diastereomic α-PHP-M1 (referred to α-PHP-M1-D1 and α-PHP-M1-D2 as defined below) as pale yellow oil. The product ratio of the diastereomers was determined by NMR spectroscopy.

Proton NMR

Proton NMR data is presented in the following order: (multiplicity, integration, coupling constants in Hz, assignment).

^{1}H NMR (400 MHz, $CDCl_3$; compound exists as a mixture of diastereomers, α-PHP-M1-D1 is denoted by §, α-PHP-M1-D2 denoted by *): *δ* 7.38–7.29 (m, 4H§ and 4H*, ArH), 7.29–7.20 (m, 1H§ and 1H*, ArH), 5.00 (d, *J* = 3.6 Hz, 1H*, -C$\underline{H}$(OH)-), 4.18 (d, *J* = 9.2 Hz, 1H§, -C$\underline{H}$(OH)-), 2.83–2.63 (m, 5H§ and 4H*, -C$\underline{H}_2$-N-C$\underline{H}_2$- and -CH(OH)C$\underline{H}$-§), 2.54 (dd, *J* = 8.8, 3.6 Hz, 1H*, -CH(OH)C$\underline{H}$-), 1.85–1.75 (m, 4H* and 4H§, -NCH_2C$\underline{H}_2$C$\underline{H}_2$-), 1.54-1.36 (m, 1H* and 1H§, -C(OH)CHC$\underline{H}_2$-), 1.33–0.92 (m, 5H* and 5H§, -C(OH)CHC$\underline{H}_2$- and -(C$\underline{H}_2$)$_2$$CH_3$), 0.74 (t, *J* = 7.2 Hz, 3H*, -CH_2C$\underline{H}_3$), 0.70 (t, *J* = 7.2 Hz, 3H§, -CH_2C$\underline{H}_3$). The ratios of diastereomers is 1.0 (D1/D2). High-resolution mass spectrometry (HRMS): calculated for $[M+H]^+$ 248.2009, found 248.2007.

α-(2″-Oxo-pyrrolidino)hexanophenone (α-PHP-M2)

To a solution of 2-bromo-hexanophenone (200 mg) in THF at 0 °C was added, dropwise, suspension of 2-pyrrolidinone and NaH (60 %, dispersion in paraffin, Tokyo Chemical Industry, Tokyo, Japan) in THF. The mixture was stirred at 0 °C for 1 h. The reaction mixture was added water and extracted with ethyl acetate. The organic extract was washed with brine, dried over anhydrous Na_2SO_4, and evaporated under vacuum. The resultant residue was subjected to column chromatography using a silica gel column and an ethyl acetate/*n*-hexane mixture (2:3, v/v) as an eluent to isolate α-PHP-M2. Finally, the solution was concentrated under vacuum to give α-PHP-M2 as pale yellow oil.

Proton NMR

^{1}H NMR (400 MHz, CD_3OD): *δ* 8.07–8.02 (m, 2H, ArH), 7.60–7.55 (m, 1H, ArH), 7.49–7.44 (m, 2H, ArH), 5.64 (dd, *J* = 9.4, 5.8 Hz, 1H, -COC$\underline{H}$$CH_2$-), 3.36 (ddd, *J* = 9.6, 8.4, 6.4 Hz, 1H, -C$\underline{H}_2$NCO-), 3.26 (ddd, *J* = 9.6, 8.4, 5.6 Hz, 1H, -C$\underline{H}_2$NCO-), 2.43 (ddd, *J* = 17, 9.6, 7.2 Hz, 1H, -NCOC$\underline{H}_2$-), 2.33 (ddd, *J* = 17, 9.2, 6.4 Hz, 1H, -NCOC$\underline{H}_2$-), 2.04–1.84 (m, 3H, -$NCOCH_2$C$\underline{H}_2$- and -COCHC$\underline{H}_2$-), 1.80–1.69 (m, 1H, -COCHC$\underline{H}_2$-), 1.49–1.15 (m, 4H, -C$\underline{H}_2$C$\underline{H}_2$$CH_3$), 0.90 (t, *J* = 7.2 Hz, 3H, -CH_2C$\underline{H}_3$). HRMS: calculated for $[M+H]^+$ 260.1645, found 260.1649.

α-PHPP and α-(2″-oxo-pyrrolidino)heptanophenone (α-PHPP-M2)

α-PHPP and α-PHPP-M2 were synthesized via α-PHP 2-bromo-heptanophenone, according to the method described above for α-PHP and α-PHP-M2, respectively.

Proton NMR of α-PHPP-M2

^{1}H NMR (400 MHz, CD_3OD): *δ* 8.07–8.02 (m, 2H, ArH), 7.60–7.54 (m, 1H, ArH), 7.50–7.44 (m, 2H, ArH), 5.64 (dd, *J* = 9.2, 5.6 Hz, 1H, $-COC\underline{H}CH_2-$), 3.36 (ddd, *J* = 9.6, 8.4, 6.0 Hz, 1H, $-C\underline{H}_2NCO-$), 3.26 (ddd, *J* = 9.6, 8.4, 5.4 Hz, 1H, $-C\underline{H}_2NCO-$), 2.43 (ddd, *J* = 17, 9.4, 7.2 Hz, 1H, $-NCOC\underline{H}_2-$), 2.33 (ddd, *J* = 17, 9.4, 6.4 Hz, 1H, $-NCOC\underline{H}_2-$), 2.04–1.84 (m, 3H $-NCOCH_2C\underline{H}_2-$ and $-COCHC\underline{H}_2-$), 1.79–1.68 (m, 1H, $-COCHC\underline{H}_2-$), 1.41–1.21 (m, 6H, $-C\underline{H}_2C\underline{H}_2\ C\underline{H}_2CH_3$), 0.88 (t, *J* = 6.6 Hz, 3H, $-CH_2C\underline{H}_3$). HRMS: calculated for $[M+H]^+$ 274.1802, found 274.1801.

1-Phenyl-2-(pyrroridin-1-yl)heptan-1-ol (α-PHPP-M1)

α-PHPP-M1 was synthesized from α-PHPP according to the above mentioned method for α-PHP-M1.

Proton NMR

^{1}H NMR (400 MHz, $CDCl_3$; compound exists as a mixture of diastereomers, α-PHPP-M1-D1 is denoted by §, α-PHPP-M1-D2 denoted by *): *δ* 7.39–7.29 (m, 4H§ and 4H*, ArH), 7.28–7.20 (m, 1H§ and 1H*, ArH), 5.00 (d, *J* = 4.0 Hz, 1H*, $-C\underline{H}(OH)-$), 4.18 (d, *J* = 9.2 Hz, 1H§, $-C\underline{H}(OH)-$), 2.82–2.62 (m, 5H§ and 4H*, $-C\underline{H}_2-N-C\underline{H}_2-$ and $-CH(OH)C\underline{H}-^§$), 2.54 (dd, *J* = 8.8, 4.0 Hz, 1H*, $-CH(OH)C\underline{H}-$), 1.88–1.75 (m, 4H* and 4H§, $-NCH_2C\underline{H}_2C\underline{H}_2-$), 1.53–1.36 (m, 1H§ and 1H*, $-C(OH)CHC\underline{H}_2-$), 1.35–0.90 (m, 7H§ and 7H*, $-C(OH)CHC\underline{H}_2-$ and $-(C\underline{H}_2)_2CH_3$), 0.77 (t, *J* = 7.2 Hz, 3H*, $-CH_2C\underline{H}_3$), 0.74 (t, *J* = 7.2 Hz, 3H§, $-CH_2C\underline{H}_3$). The ratios of diastereomers is 1.2 (D1/D2). HRMS: calculated for $[M+H]^+$ 262.2165, found 262.2163.
