# Supplementary material for: Metabolism of α-PHP and α-PHPP in humans and the effects of alkyl chain lengths on the metabolism of α-pyrrolidinophenone-type designer drugs
Source: Forensic Toxicol. 2018 May 28;36(2):486–97. doi: 10.1007/s11419-018-0428-7 (PMC6002437; doi:10.1007/s11419-018-0428-7)
Supplement: Supplementary file 2 — Supplementary material 2 (PDF 171 kb) [file 11419_2018_428_MOESM2_ESM.pdf]

Supplementary material 2:

Table S1 Validation data for the LC–MS/MS procedure established in the present study

Validation item	α-PHP	α-PHP-M1-D1	α-PHP-M1-D2	α-PHP-M2	α-PHPP	α-PHPP-M1-D1	α-PHPP-M1-D2	α-PHPP-M2
Recovery (%) [a]								
5 ng/mL	87.1	98.6	97.1	111	93.7	112	113	117
40 ng/mL	91.7	97.1	98.3	104	89.8	105	102	121
200 ng/mL	95.1	101	103	109	92.2	103	103	119
Linearity [b]								
Range (ng/mL)	3.0–300	3.0–300	3.0–300	3.0–300	3.0–300	3.0–300	3.0–300	3.0–300
Equation	$y = 0.162\,x - 0.00063$	$y = 0.598\,x + 0.00202$	$y = 0.098\,x - 0.00014$	$y = 0.298\,x - 0.00243$	$y = 0.196\,x - 0.00103$	$y = 0.519\,x - 0.00269$	$y = 0.094\,x + 0.00018$	$y = 0.186\,x - 0.00306$
R^2	0.9988	0.9986	0.9984	0.9967	0.9981	0.9984	0.9941	0.9930
LOD (ng/mL)	0.45	0.15	0.75	0.24	0.30	0.15	0.90	0.45
LLOQ (ng/mL)	1.5	0.50	2.5	0.80	1.0	0.50	3.0	1.5
Intraday accuracy (%) [c]								
5 ng/mL	+4.51	−4.81	−2.38	−4.01	−7.88	−2.56	+0.44	−6.14
40 ng/mL	−3.71	+1.72	+3.30	−6.86	−6.19	−7.09	+0.41	−14.15
200 ng/mL	−3.95	−0.60	+6.55	−3.10	−7.74	−3.07	+4.11	−5.56
Intraday precision (%) [c]								
5 ng/mL	11.94	4.33	7.15	6.27	5.23	5.81	8.13	6.00
40 ng/mL	7.45	3.39	11.01	5.07	8.49	4.60	10.44	2.91
200 ng/mL	7.09	4.13	7.35	4.00	4.55	3.34	6.26	9.36
Interday accuracy (%) [d]								
5 ng/mL	+2.84	−3.95	+2.54	+1.59	−2.91	+2.98	+3.28	−2.52
40 ng/mL	−1.29	+3.80	+5.28	−3.81	−5.68	+3.35	+2.04	−8.90
200 ng/mL	−1.33	+3.56	+5.03	+1.97	−5.60	+2.63	+8.32	+4.97
Interday precision (%) [d]								
5 ng/mL	7.69	7.71	11.43	7.52	8.83	8.79	11.06	9.50
40 ng/mL	9.82	8.26	9.42	9.07	6.63	9.40	9.77	14.37
200 ng/mL	7.65	7.39	7.22	8.67	5.83	5.36	6.87	14.10

R^2 coefficient of determination, *LOD* limit of detection, *LLOQ* lower limit of quantification (10σ)

[a] The recoveries were calculated by comparing peak areas of the analytes extracted from spiked samples with those spiked in urinary extract ($n = 5$)

[b] Five different concentrations (3 replicates per level)

[c] $n = 5$

[d] $n = 15$ (3 days, $n = 5$)
